# A novel nanoparticle vaccine, based on S1-CTD, elicits robust protective immune responses against porcine deltacoronavirus

**DOI:** 10.1128/jvi.00674-25

**Published:** 2025-08-21

**Authors:** Qing He, Yawen Zou, Beilei Yu, Qian Yuan, Chenguang Meng, Chenxuan Du, Zhiyong Wang, Jiahao Lian, Shile Luo, Siyu Cao, Wenbing Yang, Dantong Li, Hongyu Lei, Yang Zhan, Wenfeng Zhou, Yi Yang, Naidong Wang

**Affiliations:** 1 Hunan Provincial Key Laboratory of Protein Engineering in Animal Vaccines, Laboratory of Functional Proteomics (LFP) & Research Center of Reverse Vaccinology (RCRV), College of Veterinary Medicine, Hunan Agricultural University 12575, Changsha, China; Loyola University Chicago - Health Sciences Campus, Maywood, Illinois, USA

**Keywords:** porcine deltacoronavirus, S1-CTD, nanoparticle vaccine, AP205, passive immunity

## Abstract

Porcine deltacoronavirus (PDCoV), an emerging swine pathogen causing severe enteric disease in piglets, poses cross-species transmission risks, including humans. The C-terminal domain (CTD) of its spike protein harbors a key receptor-binding region with antigenic specificity and cross-protective potential. Here, we developed a novel nanoparticle-based CTD vaccine (CTDnps) by conjugating PDCoV S1-CTD to bacteriophage AP205 capsids, leveraging subunit vaccine advantages in safety, scalability, and affordability, to address urgent needs for PDCoV control. Compared to the CTD monomer, the CTDnps vaccine induced a markedly faster antigen-specific IgG response and greater neutralizing antibody (NAb) titers in mice. In immunized sows, the CTDnps vaccine elicited sustained IgG and secretory IgA (sIgA) responses, with serum NAbs persisting up to 60 days post-farrowing at titers exceeding 1:64. Passive transfer of maternal antibodies to newborn piglets significantly reduced viral loads and clinical signs upon PDCoV challenge, whereas histopathological and immunohistochemical analyses confirmed reduced viral presence and intestinal damage in the CTDnps-vaccinated group. Furthermore, CTDnps enhanced dendritic cell antigen uptake and upregulated expression of major histocompatibility complex II and co-stimulatory molecules (CD80 and CD86) to activate humoral and cellular immunity. We concluded that the PDCoV S1-CTD nanoparticle vaccine has much potential for robust and prolonged PDCoV prevention. Finally, the formed nanoparticle platform has potentially broad applications for developing multivalent vaccines against diverse coronaviruses.

**IMPORTANCE:**

Although porcine deltacoronavirus (PDCoV) poses a potential threat to public health, effective vaccines against PDCoV remain lacking. Here, we developed a novel nanoparticle-based C-terminal domain vaccine (CTDnps) targeting the conserved S1-CTD domain of the PDCoV spike protein. Unlike traditional subunit vaccines, CTDnps displayed AP205 capsids to enhance antigen presentation, induced rapid and robust neutralizing antibodies in sows, and conferred passive immunity to piglets via maternal antibody transfer. Mechanistically, CTDnps promoted dendritic cell activation and cellular immunity by upregulating major histocompatibility complex II and co-stimulatory molecules, a feature absent in monomeric CTD vaccines. We not only established CTDnps as a potent PDCoV intervention, but also pioneered a scalable, fast platform adaptable to emerging or multivalent coronavirus vaccines. This study provided actionable strategies to mitigate PDCoV outbreaks and broader coronavirus threats.

## INTRODUCTION

Coronaviruses are single-stranded enveloped RNA viruses that have caused human and many animal epidemics (1, 2). For example, COVID-19 was a global pandemic caused by SARS-CoV-2 (3). Porcine deltacoronavirus (PDCoV), a highly contagious porcine enteric coronavirus belonging to the genus *Deltacoronavirus*, was first discovered in 2009 in Hong Kong, China, with confirmed spread to mainland China, South Korea, Southeast Asia, and North America (4–6). PDCoV infects pigs of all ages, with newborn piglets being the most susceptible, with clinical signs including severe acute diarrhea, vomiting, dehydration, and even death of piglets (7, 8). Compared to PEDV and TGEV, PDCoV has a “unique” pathogenicity that includes infecting the lungs and causing mild interstitial pneumonia in piglets (9).

The PDCoV S protein is a class I transmembrane trimer glycoprotein with an N-terminal S1 domain responsible for receptor binding and a C-terminal S2 domain responsible for membrane fusion. The S1 domain is further divided into four distinct regions: NTD, CTD, SD1, and SD2 (10). NTD binds to the sialic acid receptor on the cell surface, whereas CTD has been involved in binding to the main entry receptor aminopeptidase N (APN) (11–13).

PDCoV has cross-species transmission, infecting pigs, calves, chickens, birds, mice, and even humans (4, 14–17). PDCoV can utilize various APNs from eight species as functional receptors to enter non-susceptible cells (18, 19). Furthermore, the CTD domain of the PDCoV S1 subunit directly establishes distinct receptor-binding modes with various APNs (11, 19). Moreover, monoclonal antibody 67B12, which bound to the APN-binding CTD domain, had a virus-neutralizing role by occupying the binding site between CTD and APN (20). It is noteworthy that CTD had a stronger affinity when combined with human APN than porcine APN (11). Recent evidence demonstrated that the CTD domain functions as a receptor-binding domain (RBD) in viral transmission and infection, further exposing PDCoV to cross-species transmission risk.

Although PDCoV is a potentially zoonotic virus, no effective vaccine is currently available. Among all types of vaccines, subunit vaccines have good safety and flexible scalability (21, 22). The RBD of the coronavirus spike protein, which contains the critical neutralizing domain, is a promising target for vaccine development (23–25). Moreover, RBD-based vaccines provide cross-protection against diverse coronavirus strains and also offer advantages in terms of production scalability, stability, and affordability (26–28). Currently, it is believed that the RBD of PDCoV is located in the S1-CTD domain of the S protein, the major antigenic component of neutralizing antibody (NAb) (11, 20, 29, 30).

Virus-like particles (VLPs) are good candidates for vaccine development, as they can be readily modified by genetic engineering or chemical conjugation (31, 32). VLPs have many advantages (e.g., safety and strong immunogenicity) that enable them to be used as vaccines or as platforms for vaccine design. They are nanoparticles with diameters of 20–200 nm, enabling them to efficiently enter the lymphatic system and/or to be taken up by antigen-presenting cells (APCs) (33). Moreover, coat proteins on the capsid are displayed in a multi-valent manner, which promotes activation of B cells by cross-linking B-cell receptors (34–38). Bacteriophage AP205, an icosahedral capsid with a diameter of 30 nm and composed of 180 subunits, has been successfully used for the unidirectional, high-density display of diverse foreign proteins on the surface (e.g., HBV E2, HIV gp140, and H1N1 M2e) to significantly boost both humoral and cellular immune responses (39–45). Compared to VLPs derived from human and animal viruses, AP205 VLPs have many advantages: (i) humans and animals lack preexisting antibodies against AP205 VLPs, which can attenuate immunogenicity of the platform (45); (ii) AP205 VLPs have 180 subunits, which can display more foreign antigens on the surface than other VLPs; and (iii) bacteriophage VLPs can be manufactured cheaply and in large quantities in bacteria, compared to eukaryotic expression systems.

This platform has already been used to develop candidate vaccines to protect humans from SARS-CoV-2, malaria, and West Nile virus (39, 44, 46, 47). Immunization with ABNCoV2a, a SARS-CoV-2 RBD-conjugated AP205 nanoparticle vaccine, significantly reduced viral load in monkeys and was approved for Phase I clinical trials (48). Similarly, immunization with AP205 VLPs displaying domain III from West Nile virus completely protected mice against virus challenge (49). However, there has been limited application of this platform for the development of vaccines for use in animals.

Herein, designed CTD-AP205 nanoparticles (CTDnps), using antigens and particle carriers in optimal proportions, were constructed by displaying S1-CTD antigens of PDCoV on the surface via a covalent ligation strategy of the SpyTag-SpyCatcher system. Pregnant sows were immunized to assess passive immune protection for piglets, mediated by the CTDnps vaccine, against a PDCoV challenge. Uptake and presentation of nanoparticles by dendritic cells (DCs) were further investigated. The objective was to explore strategies to enhance immunoprotection of PDCoV S1-CTD as a promising candidate vaccine and to provide a basis for designing vaccines with rapidly interchangeable antigens and improved efficacy against PDCoV and other coronaviruses.

## RESULTS

### Design of self-assembled nanoparticle vaccine against PDCoV

**Fig 1:**
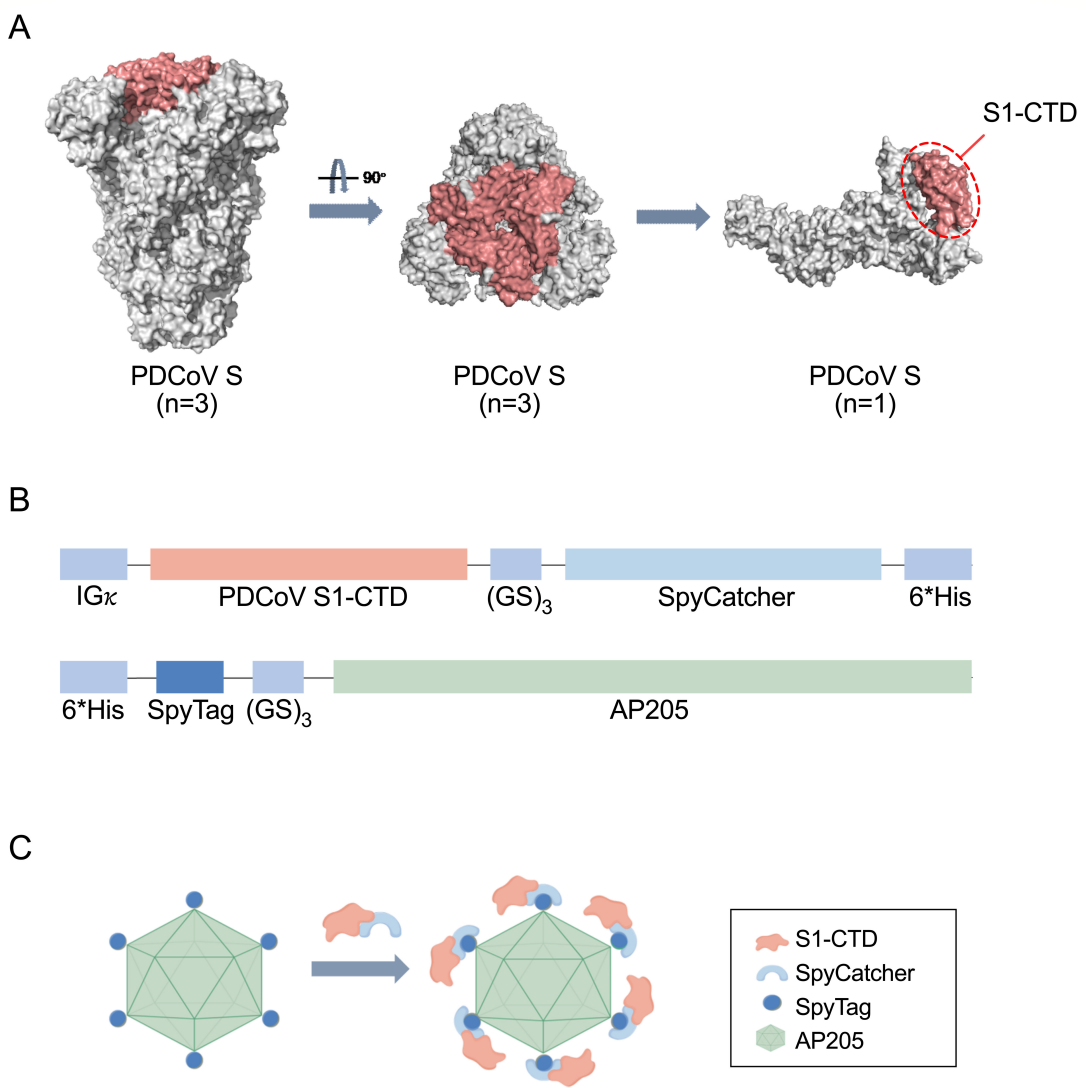
Design and construction of CTD-AP205 nanoparticles (CTDnps). (**A**) Overall structure of CTD in the PDCoV S ectodomain. (**B**) Construction of CTD and AP205 protein expression plasmids for HEK293F and *E. coli* expression systems. (**C**) CTD-SpyCatcher covalently bound to SpyTag-AP205 to form CTDnps.

The three S1-CTDs were located at the top and center of the spike trimer (Fig. 1A). Self-assembled nanoparticles of S1-CTD (300–420 aa) of PDCoV were designed by displaying antigens on the surface of self-assembled AP205 nanoparticles via a covalent ligation strategy of the SpyTag-SpyCatcher system. S1-CTD genes were synthesized according to the codon preference of mammalian expression, and the IGκ signal peptide sequence was added to ensure protein secretion. S1-CTD and SpyCatcher (SC) were joined via the flexible glycine-serine linker (GS)_3_, with a 6×His tag added to the end of the CTD-SC recombinant genes. According to the codon preference of *Escherichia coli* (*E. coli*), synthesized AP205 genes were linked to the 3′ end of the fusion gene of the flexible glycine-serine linker (GS)_3_-SpyTag (ST), downstream of the 6×His tag, forming ST-AP205 recombinant genes (Fig. 1B). Purified CTD-SC and ST-AP205 were mixed and incubated to form CTDnps (Fig. 1C).

### Characterization of CTD-AP205 nanoparticles

**Fig 2:**
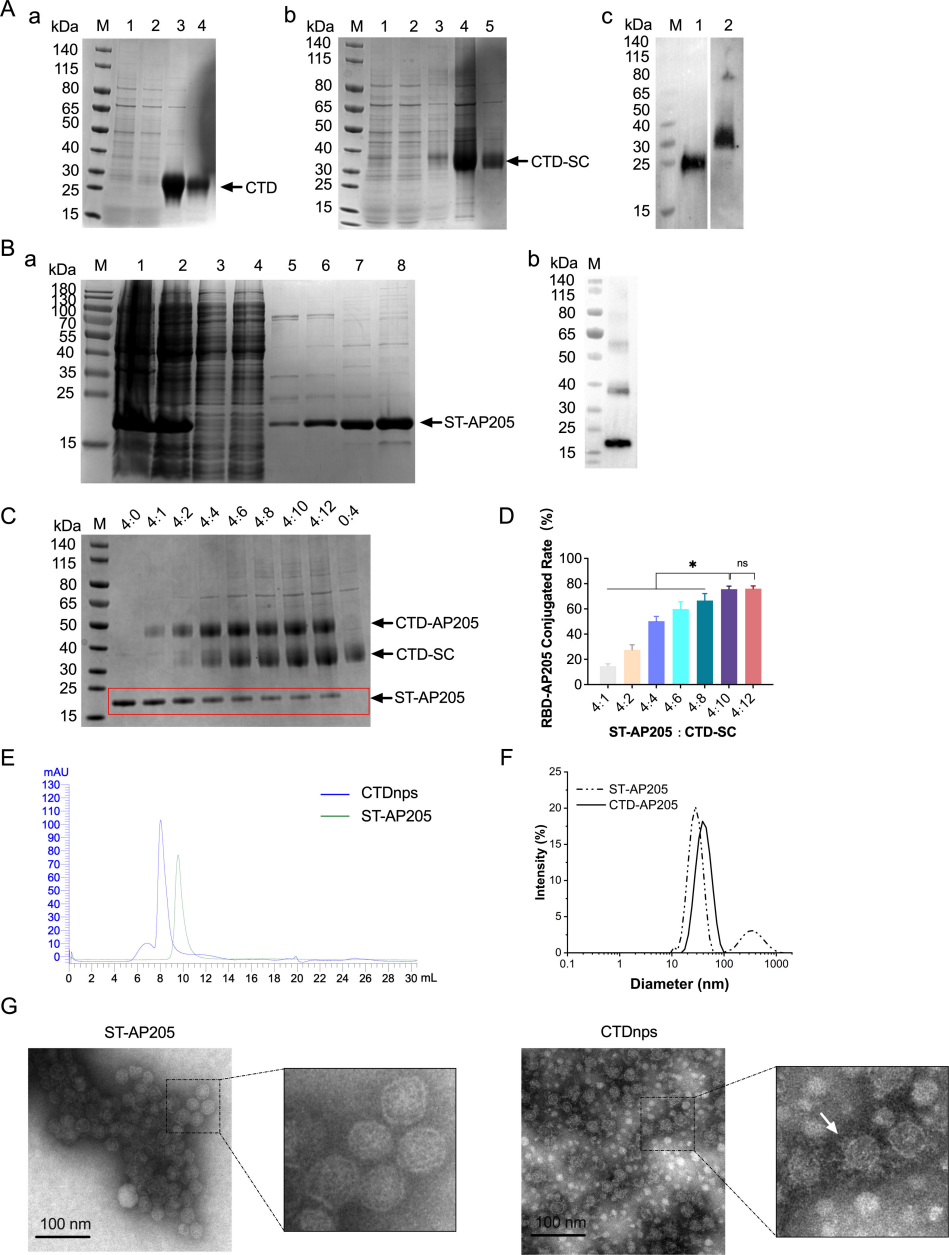
Preparation and characterization of recombinant protein. (**A**) (a) Expression and purification of CTD proteins. M: protein labeling; Lane 1: whole-cell lysate; Lane 2: supernatant; and Lanes 3 and 4: purified protein. (b) Expression and purification of CTD-SC proteins. Lane 1: whole-cell lysate; Lane 2: supernatant; Lane 3: wash sample; and Lanes 4 and 5: purified protein. (c) Purity of CTD and CTD-SC proteins was assessed using western blot with anti-PDCoV polyclonal antibody. Lane 1: CTD and Lane 2: CTD-SC. (**B**) (a) Expression and purification of ST-AP205 proteins. M: protein labeling; Lanes 1–8 represent whole bacteria, supernatant, flow through, wash, and purified protein. (b) Purification of ST-AP205 proteins was analyzed using western blot with anti-His monoclonal antibody. (**C and D**) Detection of the SDS-PAGE assembly of CTD-SC and ST-AP205 nanoparticles mixed under various molar ratio conditions. (**E**) SEC of unconjugated nanoparticles (ST-AP205) and CTDnps. (**F**) DLS analysis of the unconjugated nanoparticles (ST-AP205) and CTDnps. (**G**) TEM of unconjugated nanoparticles (ST-AP205) and CTDnps.

Recombinant proteins were obtained using a His-tag-based Ni-NTA column and subjected to SDS-PAGE and western blotting; recombinant proteins of CTD, CTD-CS, and ST-AP205 had molecular weights of ~28, 40, and 20 kDa, respectively (Fig. 2A and B). Based on binding analysis, CTD-SC recombinant proteins bound specifically with the ST-AP205 recombinant proteins through covalent ligation between the SpyTag and SpyCatcher, forming CTD-AP205 nanoparticles (CTDnps) complexes (Fig. 2C). The rate of formation of CTDnps was >80% when the molar ratio between the ST-AP205 recombinant proteins and CTD-SC recombinant proteins was 4:10 (Fig. 2D). Based on size-exclusion chromatography (SEC) purification, CTDnps were purified at ∼8 mL elution, whereas unconjugated nanoparticles (ST-AP205) were purified at ∼10 mL elution (Fig. 2E). When hydrodynamic diameters of ST-AP205 and CTDnps were measured and analyzed by dynamic light scattering (DLS), ST-AP205 and CTDnps had particle sizes of 28 and 36 nm, respectively (Fig. 2F). Based on transmission electron microscopy (TEM), there were regular small protrusions on the surface of CTDnps when compared to ST-AP205 (Fig. 2G).

### Immunogenicity of CTD monomer and CTD nanoparticles in BALB/c mice

**Fig 3:**
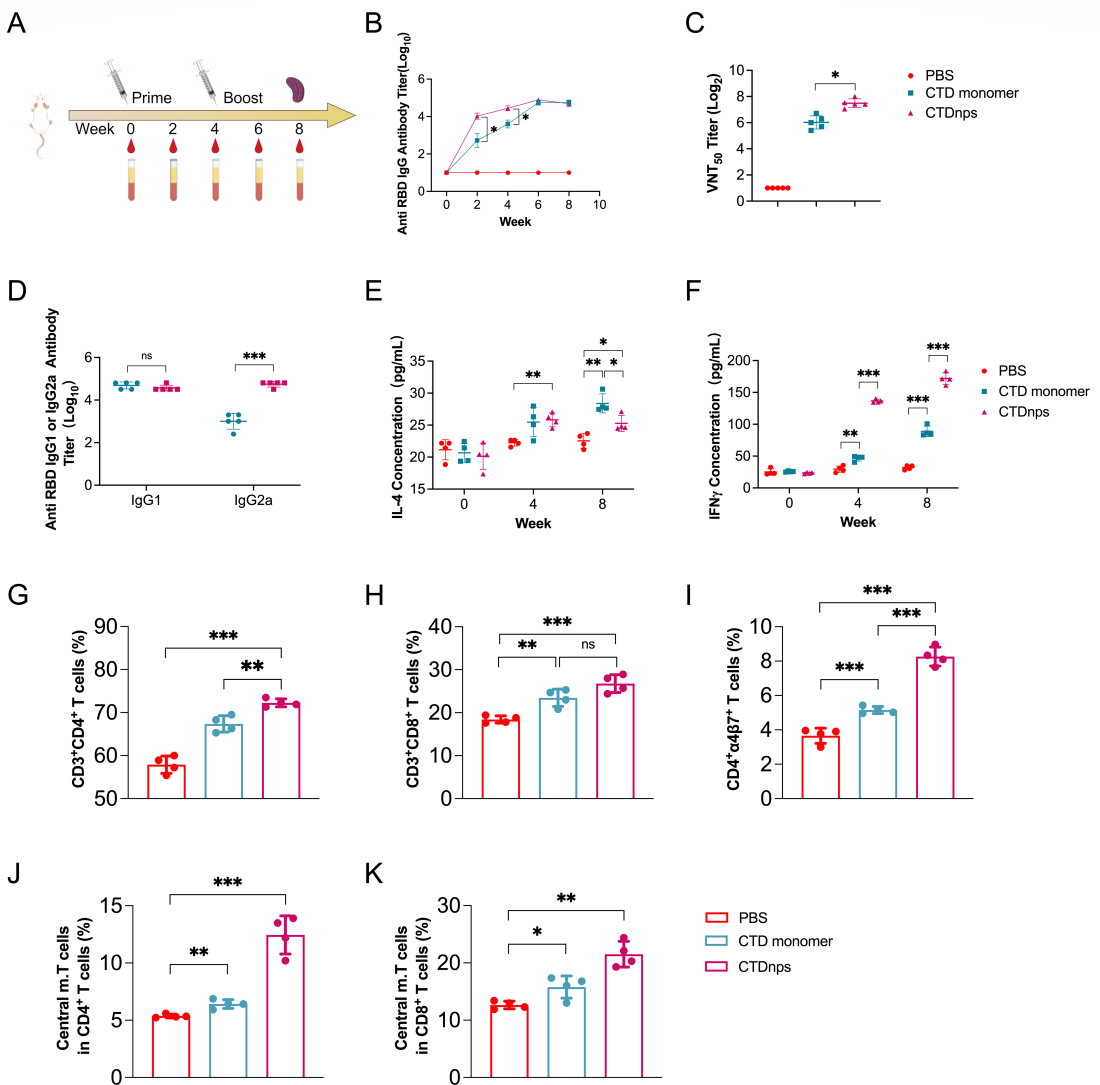
CTDnps vaccine elicited robust antibody and T cell responses in mice. (**A**) Schematic diagram of the mice immunization procedure. Five mice per group received prime/boost vaccination with various vaccines at Weeks 0 and 4. Serum samples were collected every 2 weeks. (**B**) Titers of antigen-specific IgG antibodies were measured in serum samples by iELISA. (**C**) Titers of NAbs were measured in serum samples from mice at Week 8 by VNT. (**D**) Titers of antigen-specific IgG1 and IgG2a for each immunization group. Serum samples from mice at Week 8 were tested. IL-4 (**E**) and IFN-γ (**F**) concentrations were measured by double-antibody sandwich ELISA. (**G, H**) The percentage of CD3^+^CD4^+^ and CD3^+^CD8^+^ T lymphocytes among spleen lymphocytes was determined by flow cytometry. (**I**) The percentage of CD4^+^α4β7^+^ T lymphocytes among spleen lymphocytes was determined by flow cytometry. (**J, K**) The percentage of CD4^+^ and CD8^+^ Tcm among spleen lymphocytes was determined by flow cytometry. Data are mean ± SD. Asterisks indicate significant differences (*, *P* < 0.05; **, *P* < 0.01; ***, *P* < 0.001; and ns, *P* > 0.05).

To evaluate immunogenicity elicited by CTDnps, three groups of BALB/c mice (*n* = 5) were immunized two times with CTD, CTDnps, or PBS at Weeks 0 and 4, respectively (Fig. 3A). An indirect enzyme-linked immunosorbent assay (iELISA) was used to determine whether CTD nanoparticles promoted humoral immunity. After the priming vaccination, CTDnps rapidly elicited higher levels of antigen-specific IgG than CTD monomer at Weeks 2 and 4 (Fig. 3B); however, IgG titers were not significantly different between CTDnps and CTD monomer after the booster vaccination (Fig. 3B). A viral neutralization test (VNT) was used to measure titers of NAbs in serum samples at Week 8; titers in CTDnps mice were considerably higher than CTD monomer or PBS groups (Fig. 3C).

Antigen-specific Th1 and Th2 cell subsets promote production of various antibodies (e.g., IgG2a and IgG1) by B cells (50, 51). Both CTDnps and CTD monomer elicited equivalent levels of CTD-specific IgG1. However, the CTD-specific IgG2a induced by CTDnps was significantly higher than the CTD monomer (Fig. 3D). To assess the cellular immune response in mice vaccinated with CTDnps, concentrations of IL-4 and IFN-γ cytokines were determined by iELISA. After the primary vaccination, IL-4 concentrations were not significantly different between CTDnps and CTD monomer; however, IFN-γ concentrations elicited by CTDnps were 2.9-fold higher than that elicited by CTD monomer (Fig. 3E and F). Furthermore, after the booster vaccination, IL-4 concentrations were significantly higher in mice immunized with monomeric CTD compared to either CTDnps or PBS, whereas IFN-γ elicited by CTDnps was 2.1-fold higher than that elicited by monomeric CTD (Fig. 3E and F). Therefore, immunization with CTDnps provoked a Th1-biased cellular immune response in mice.

To further evaluate the cellular immune response in mice vaccinated with CTDnps, flow cytometry was used to quantify the T cell subsets in splenic lymphocytes. CTDnps induced more CD3^+^CD4^+^ and CD3^+^CD8^+^ T cells than CTD monomer (Fig. 3G and H). Integrin α4β7 mediates CD4^+^ T cell homing to gut-associated lymphoid tissue by binding mucosal addressin cell adhesion molecule-1 on intestinal endothelial cells (52, 53). In this study, CTDnps significantly enhanced the expression of α4β7 in CD4^+^ T cells, indicating that CTDnps has the potential to induce a stronger mucosal immune response (Fig. 3I). Flow cytometry was used to evaluate the ability of the CTDnps to elicit CD4^+^ and CD8^+^ central memory T cells (Tcm) in the spleen, crucial for the host’s antiviral response. CTDnps markedly increased the proportion of CD4^+^ and CD8^+^ central memory T cells (CD44^high^CD26L^high^) (Fig. 3J and K). Collectively, these findings confirmed that CTD nanoparticles effectively induced robust humoral and cellular immune responses in mice.

### Effective humoral immune responses were induced by CTD nanoparticles in sows

**Fig 4:**
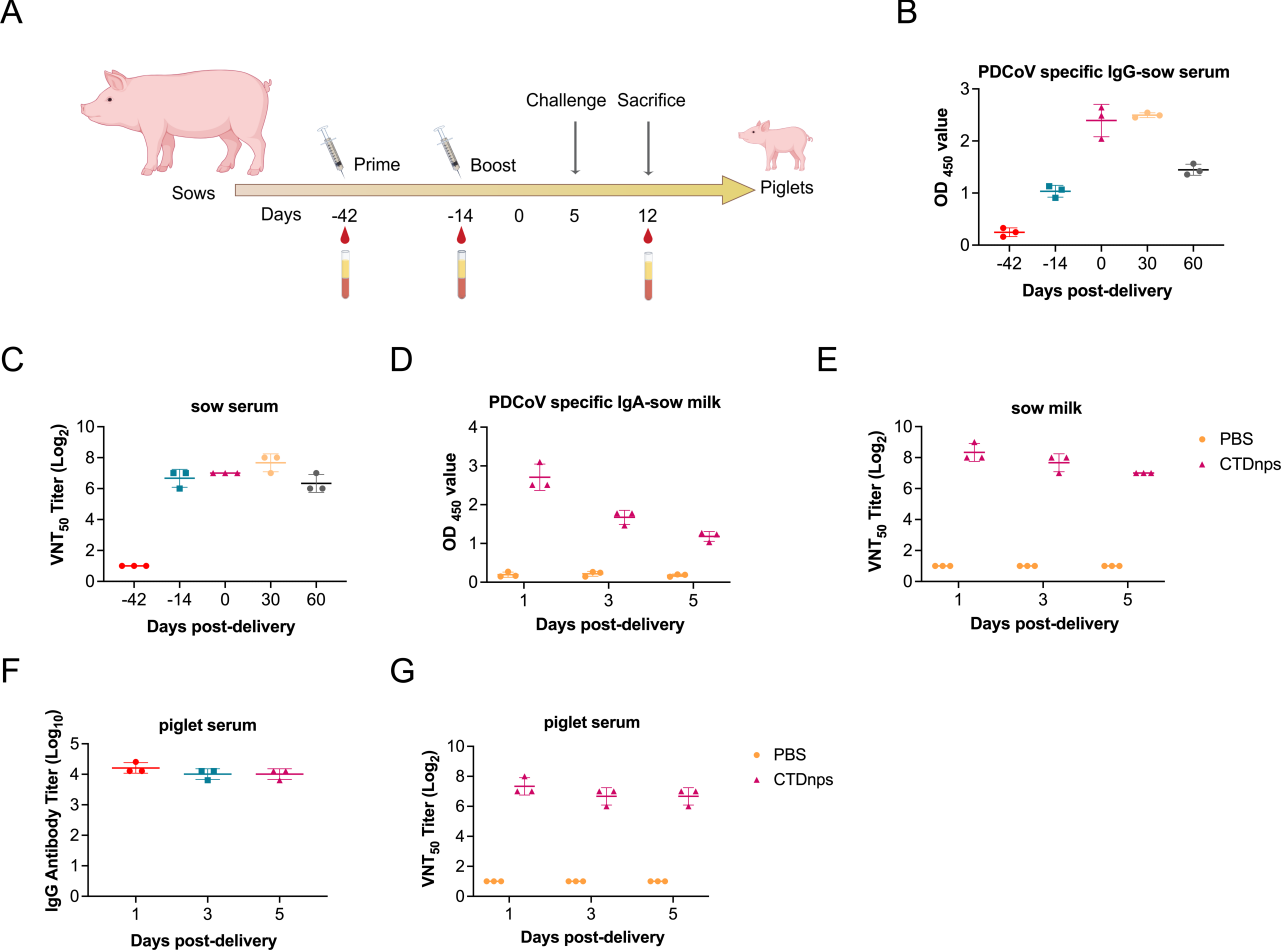
Immunogenicity of the CTDnps vaccine in piglets. (**A**) Schematic diagram of the sow’s immunization procedure. (**B**) Titers of antigen-specific IgG antibodies were measured iELISA in serum samples from sows. Titers of NAbs were measured VNT in serum (**C**) and milk (**E**) samples from sows. (**D**) Titers of antigen-specific sIgA antibodies were measured by iELISA in milk samples from sows. Titers of antigen-specific IgG antibodies (**F**) and NAbs (**G**) were measured by VNT in serum samples from newborn piglets. Data are mean ± SD. Asterisks indicate significant differences (*, *P* < 0.05; **, *P* < 0.01; ***, *P* < 0.001; and ns, *P* > 0.05).

Under field conditions, PDCoV is particularly pathogenic in newborn piglets, as their immune system is not fully developed and protection from virus infection mainly depends on maternal antibody (54). Therefore, the immune response to the CTDnps vaccine was evaluated in pregnant sows (*n* = 3), as well as its protective ability through passive immunity in piglets (Fig. 4A). CTDnps induced high PDCoV-specific IgG titers after immunization and lasted at least 60 days after farrowing (Fig. 4B). Moreover, high PDCoV-specific sIgA titers were detected in the milk of sows immunized with CTDnps, although sIgA concentrations gradually decreased over time (Fig. 4D). Based on VNT, NAbs had prolonged persistence in sow serum (reached 1:128 at 60 days post-farrowing; Fig. 4C) and sow milk (reached 1:128 at 5 days post-farrowing; Fig. 4E). The CTDnps-induced antibody levels of passive transfer antibody in the serum from sows to newborn piglets were evaluated. During the first 5 days, there were high titers of antigen-specific IgG antibodies, with NAbs in serum of newborn piglets exceeding 1:64 (Fig. 4F and G).

### CTD nanoparticles protected newborn piglets against PDCoV

**Fig 5:**
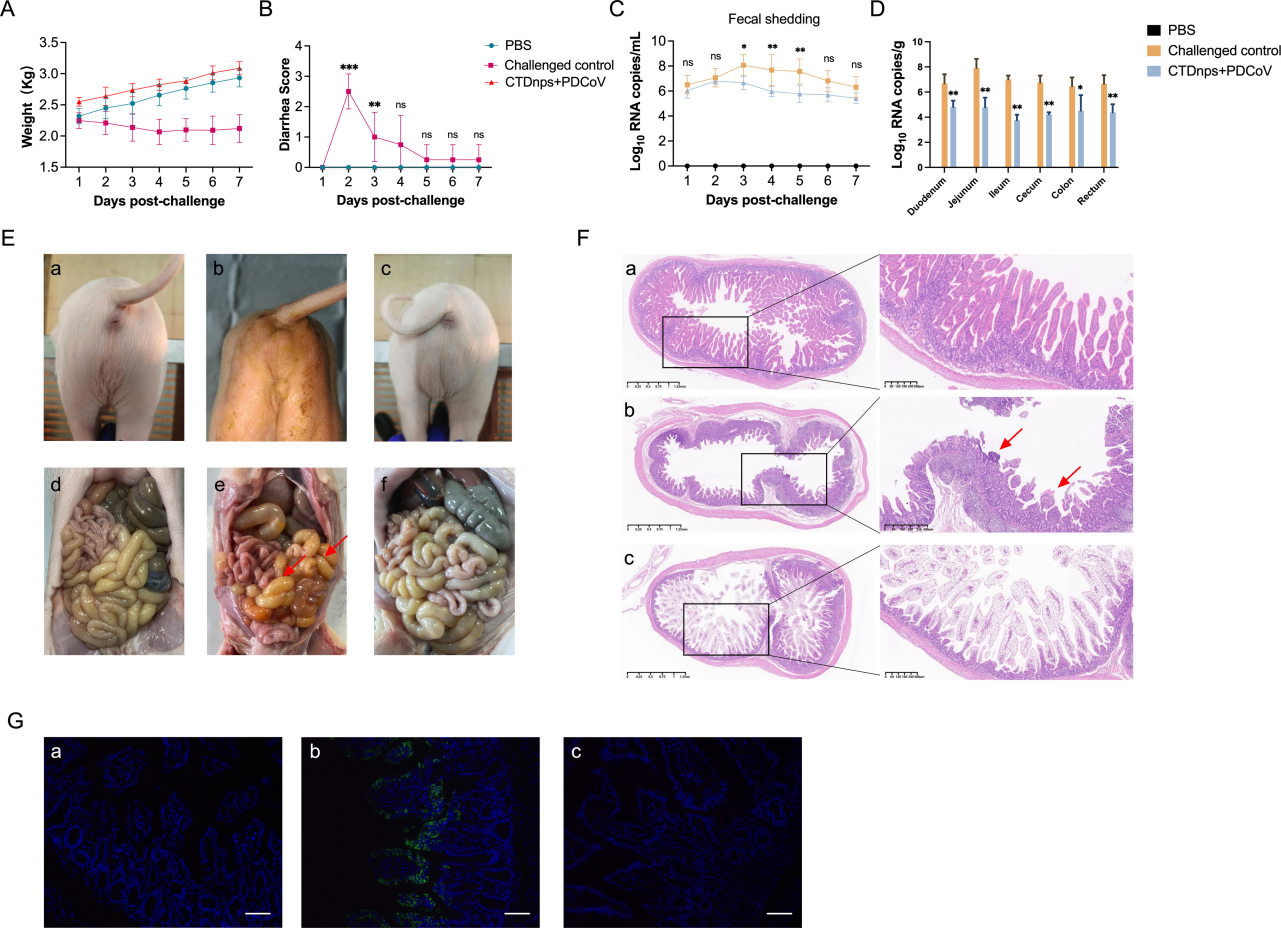
Protective immunity of the CTDnps vaccine against PDCoV in piglets. (**A**) Daily weight of piglets after challenge with PDCoV HuN/2023. (**B**) After challenge, diarrhea symptoms of each piglet were monitored and scored daily. (**C**) Piglets were inoculated with PDCoV HuN/2023, and the amount of virus shed in fecal swab was assessed daily by RT-qPCR. (**D**) Piglets were inoculated with PDCoV HuN/2023, and viral load in the duodenum, jejunum, ileum, cecum, colon, and rectum was detected by RT-qPCR after necropsy. (**E**) (a) PBS group was inoculated orally with DMEM. Challenged control group (b) and CTDnps group (c) were inoculated with PDCoV HuN/2023. All piglets in the challenged control group had watery diarrhea during the study. Gross pathology of piglets in the PBS group (d), Challenged control group (e), and CTDnps group (f). In the challenged control group, the intestinal wall of the piglets was thinner, and fluid and gas accumulated (red arrow). (**F**) Microscopic lesions of piglet’s ileum in PBS group (a), challenged control group (b), and CTDnps group (c). (**G**) PDCoV antigen was detected in the ileum of challenged control group by immunofluorescence assay. Scale bar = 100 µm. Data are mean ± SD. Asterisks indicate significant differences (*, *P* < 0.05; **, *P* < 0.01; ***, *P* < 0.001; and ns, *P* > 0.05).

Piglets (5 days old) from the CTDnps group and the PBS group were challenged with PDCoV HuN/2023 strain (4 mL × 10^5.0^ TCID_50_/mL per piglet), to evaluate protection induced by CTDnps. All piglets survived, but the challenged control group had body weight loss and transient diarrhea after challenge, whereas the PBS group and the CTDnps immunization group did not have diarrhea [Fig. 5A, B and E (a-c)]. Viral loads in fecal swabs and intestinal segments were detected by RT-qPCR. In the challenged control group, PDCoV RNA was detected in all fecal swabs collected between 1 and 6 dpi, with a peak at 3 dpi (Fig. 5C). The CTDnps immunization piglets had a significantly lower PDCoV genome copy number than the challenged control group, and no PDCoV RNA was detected in fecal swabs of the PBS control group (Fig. 5C). The distribution of PDCoV antigen in the intestinal samples was also quantified at 7 dpi. The PDCoV antigen was widely distributed in the small intestine of the challenged control group, whereas viral loads were significantly lower in the small intestine of the CTDnps immunized group (Fig. 5D).

Gross pathology and histopathology were assessed at 7 dpi. In the challenged control group, there was an accumulation of yellowish fluid in the small intestine, characterized by transparency, thin walls, and marked gas distension (Fig. 5E (e)). However, piglets from the CTDnps immunized and PBS groups had a normal intestinal appearance, with no discernible lesions in either tissues or organs (Fig. 5E (d and f)). In addition, in histopathological analyses, there was glandular necrosis in the ileum of the challenged control group and the villus was shed, fragmented, and atrophied (Fig. 5F (b)). By contrast, no obvious histopathological damage was observed in intestines of CTDnps immunized or PBS groups (Fig. 5F (a and c)). Based on the immunofluorescence assay against the PDCoV nucleocapsid protein, in the challenged control group, the PDCoV antigen was mainly distributed in the cytoplasm of infected villous epithelial cells, a phenomenon not seen in either the CTDnps-immunized or PBS groups (Fig. 5F (d-f)). Therefore, we concluded that CTDnps protected newborn piglets against PDCoV challenge.

### Analysis of CTD nanoparticles uptake and activation by DCs

**Fig 6:**
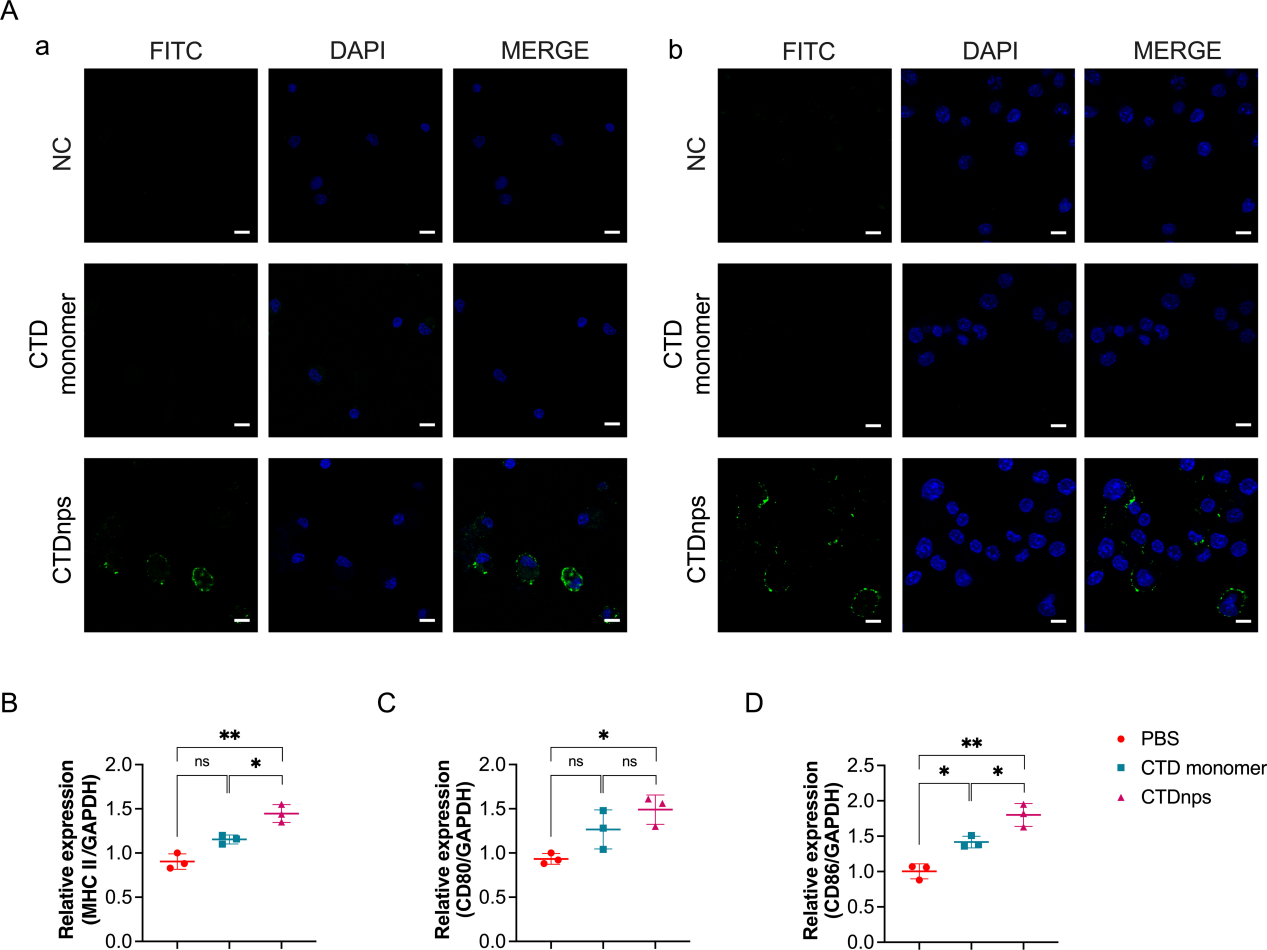
Analysis of nanoparticle protein uptake and activation by APCs. (**A**) Analysis of uptake of CTD monomer and CTDnps by BMDCs (a) or DC2.4 cells (b). Scale bar = 10 µm. The mRNA expression levels of BMDC maturation markers were assessed (RT-qPCR) based on *MHC II* (**B**), *CD80* (**C**), and *CD86* (**D**). Data are mean ± SD. Asterisks indicate significant differences (*, *P* < 0.05; **, *P* < 0.01; and ns, *P* > 0.05).

Antigen uptake mediated by APCs is the first step in initiation of vaccine-induced immune responses (55, 56). DCs are the most effective APCs for initiating T lymphocyte responses. Thus, the uptake of CTD monomer and nanoparticle by two types of DCs was measured. CTDnps were more efficient in terms of antigen uptake than the CTD monomer protein in BMDCs and DC2.4 cells (Fig. 6A). Immune activation capacity of CTDnps was further investigated in BMDCs. After co-incubating CTD protein and CTDnps with BMDCs for 6 h, mRNA expression of BMDCs maturation markers, including antigen presentation (major histocompatibility complex II [MHC II]) and co-stimulatory molecules (CD80/86), which provide obligatory signals for T-cell initiation, was assessed by RT-qPCR. The mRNA expression level of *MHC II* and *CD86* in the CTDnps group was significantly increased compared to the CTD proteins group, but there was no significant difference in *CD80* (Fig. 6B through D). Therefore, CTDnps induced not only humoral immunity but also stronger cellular immunity than non-particulate antigens.

## DISCUSSION

The emerging PDCoV has high pathogenicity in newborn piglets, causing clinical signs (mild to moderate or acute diarrhea, vomiting, dehydration, and mortality) and intestinal lesions, leading to substantial economic losses (7, 8). Unlike other swine enteric coronavirus (SeCoV), PDCoV can infect multiple cell types and animal species (4, 14–18). Specifically, PDCoV infection was detected in Haitian children, indicating that PDCoV can spread from animals to humans (16), highlighting the need for an effective vaccine to protect both swine and public health.

Current research in coronavirus vaccines primarily targets the spike (S) protein, particularly its RBD, as the main antigenic component for inducing protective immunity (26, 27). As an antigen, RBD has higher stability, broad spectrum, and ability to induce NAb production compared to whole S protein, and the specific antibody induced by vaccines based on RBD in immunized animals was 5- to 25-fold higher than that induced by S1 subunits (57–59). The PDCoV RBD is located in S1-CTD and induced NAb in mice (60, 61). However, it is unclear whether RBD induces sufficient immune protection against PDCoV infection, especially in newborn piglets.

Compared to existing PDCoV vaccines, mRNA vaccines encoding spike protein elicit the highest NAb titers in serum and milk from immunized sows against PDCoV; however, their production and distribution costs exceed those of subunit vaccines (62). PDCoV subunit vaccines, S ectodomain, S1 subunit, and VLPs all conferred good immunogenicity and immune protection in pigs (62–66). Although the immunogenicity of CTD monomer was lower than that of the S ectodomain in previous studies (64), a novel CTDnps overcame this limitation. The novel designed CTDnps vaccine enabled CTD to be displayed on the surface of AP205 particles with high density, significantly improving CTD immunogenicity (Fig. 3B and C). And CTDnps induced NAb titers in sows similar to the S ectodomain (≥1:128), despite using a lower antigen dose (50 µg per sow) (64).

Although NAbs are important for evaluating vaccine efficacy, cellular immunity is crucial for a coronavirus vaccine (e.g., SARS-CoV-2), especially for vaccines that use an RBD antigen to protect against infection (67, 68). There is recent evidence that cellular immunity has a dual role in the protection induced by an RBD vaccine. CD4^+^ T lymphocytes promote germinal center (GC) reaction by activating T follicular helper (Tfh) cells and further improve the titer and quality of NAbs induced by RBD vaccine (69, 70), whereas CD8^+^ T cells develop cytotoxic functions and generate effector memory cells to directly kill infected cells and limit virus transmission and replication (68, 71). In addition, cellular immunity has a key role in the formation of immune memory (67, 72). In this study, CTDnps markedly induced stronger CD4^+^ T and CD8^+^ T cell responses in mice (Fig. 3G through K). The robust CD4^+^ T cell response, essential for optimal B cell help and GC reactions, likely contributed to increased NAb titers.

Passive immunization can provide rapid protection, especially in acute infection and in newborn piglets that are unable to produce antibodies in the early stages of virus infection. Nanoparticle vaccines enhanced mucosal immune response and promoted sIgA secretion (73–75). In the present study, CTDnps induced a high titer and long-lasting PDCoV-specific IgG antibodies in sows, with large amounts of PDCoV-specific sIgA antibodies secreted in milk and efficiently transferred to newborn piglets. Furthermore, virus challenge in newborn piglets demonstrated protective effects of the CTDnps vaccine. The control group had body weight loss and transient diarrhea after challenge, whereas the PBS group and the CTDnps immunization group did not have diarrhea (Fig. 5A and B). Furthermore, the antigen load of PDCoV in newborn piglets from the immunization group was considerably less than that in the challenged control group. The presence of PDCoV antigen in the ileum was undetectable via immunofluorescence, with less intestinal villi damage detected histologically. Therefore, the CTDnps vaccine provided protective passive immunity against PDCoV infection in newborn piglets.

A recent study reported that two epitopes of CTD-targeting antibodies have broad ability to neutralize PDCoV variants *in vitro* (29). This further highlighted the advantages of CTDnps as a PDCoV vaccine candidate for emerging strains. Co-infections of PDCoV, PEDV, and rotavirus were frequently detected in the field, and nanoparticles displayed a variety of antigens (76–78). In future research, we will consider multiple related viruses and construct two-component updated vaccines adapted for the prevention and control of a variety of diseases.

In this study, a self-assembled PDCoV nanoparticle vaccine candidate was constructed. Compared to monomeric protein, CTDnps induced stronger T and B cell immune responses in mice. Moreover, CTDnps also protected piglets against PDCoV challenge. Our data reinforced that S1-CTD nanoparticles were an effective and safe vaccine candidate against PDCoV.

## MATERIALS AND METHODS

### Cells and viruses

HEK293F cells (Thermo Fisher Scientific, Waltham, MA, USA) were cultured in suspension using serum-free SMM 293-TII medium (Sino Biological Inc., Beijing, China) at 37°C with 5% CO_2_. Bone marrow-derived dendritic cells (BMDCs) were isolated from the femurs of 6-week-old healthy native pigs, free of PCV2, PRRSV, porcine parvovirus, and other major swine pathogens were cultured in RPMI-1640 (Gibco, Grand Island, NY, USA) supplemented with 10% fetal bovine serum, 0.1% antibiotics, 5 ng/mL recombinant porcine GM-CSF (R&D Systems, Minneapolis, MN, USA), and 20 ng/mL recombinant porcine IL-4 (R&D Systems) at 37°C with 5% CO_2_. The medium was changed every 2 days, and immature BMDCs were harvested on day 7 and used for further experiments. DC2.4 cells were stored in our laboratory and cultured at 37°C with 5% CO_2_ in RPMI-1640 supplemented with 10% fetal bovine serum and 0.1% antibiotics. The PDCoV HuN/2023 strain (GenBank accession number: PQ753145) was isolated from a piglet with diarrhea and stored in our laboratory.

### Plasmid construction

Nucleic acid sequences of the PDCoV S1-CTD (amino acid residues 300–420) and S1-CTD-SpyCatcher were codon-optimized for eukaryotic cells and synthesized by GenScript (Nanjing, China). Recombined genes were cloned into the pcDNA3.4 expression vector with an N-terminal IG*κ* signal peptide and C-terminal 6×His tag. Similarly, nucleic acid sequences of the AP205 coat protein (GenBank accession number: NP085472) were codon-optimized for *E. coli* and synthesized by GenScript. Recombined genes were cloned into the pET28a expression vector with an N-terminal 6×His tag and SpyTag (AHIVMVDAYKPTK).

### Protein expression and purification

To express SpyTag-AP205 protein, pET28a-ST-AP205 plasmids were transformed into *E. coli* BL21 competent cells that were grown for 12 h at 37°C on LB agar plates containing 50 µg/mL kanamycin. A single positive colony was transferred into 10 mL LB medium containing 50 µg/mL kanamycin and incubated overnight at 37°C with shaking at 200 rpm. Next, the cultures were added into 1 L fresh LB medium containing 50 µg/mL kanamycin and incubated at 37°C, 200 rpm with shaking. When the culture reached an optical density (OD) of ~0.6–0.8 at 600 nm, isopropyl-β-d-thiogalactopyranoside was added (final concentration, 0.5 mM) and induction was continued for 16 h at 28°C at 200 rpm with shaking. Bacteria were collected by centrifugation (5,000 × *g* for 10 min at 4°C) and resuspended in 40 mL of lysis buffer (25 mM Tris-HCl, 300 mM NaCl, and 10 mM imidazole, pH = 8.0). Sonication was performed on ice, and the supernatant was collected after centrifugation (20,000 × *g* for 20 min at 4°C). Supernatants were incubated with Ni-NTA Agarose (QIAGEN, Duesseldorf, Germany) at 4°C for 1 h and washed with five column volumes of buffer (25 mM Tris-HCl, 300 mM NaCl, and 30 mM imidazole, pH = 8.0) by gravity filtration. The target protein was eluted with elution buffer (25 mM Tris-HCl, 150 mM NaCl, and 300 mM imidazole, pH = 8.0) and concentrated using a 100 kDa MWCO ultra-centrifugal filter (Millipore, Billerica, MA, USA).

To express S1-CTD and S1-CTD-SpyCatcher proteins, HEK293F cells (2 × 10^6^ cells/mL) were individually transfected with pcDNA3.4-IG*κ*-CTD and pcDNA3.4-IG*κ*-CTD-SC plasmids. After 4 days of incubation, the protein was harvested from cell culture medium and purified through Ni-NTA affinity chromatography, as described above. Purified proteins were concentrated and buffer-exchanged into PBS by a 10 kDa MWCO ultra-centrifugal filter (Millipore). All proteins were stored at −80°C.

### CTD-SC and ST-AP205 conjugations

Purified ST-AP205 and CTD-SC recombinant proteins were mixed with designated molar ratios and left overnight at 4°C. SDS-PAGE was performed to measure covalent coupling between ST-AP205 and CTD-SC. To separate the CTDnps complex from the CTD-SC and ST-AP205 mixed samples, samples were purified using SEC on a Superdex 200 Increase 10/300 Gl column (Cytiva, MA, USA) pre-equilibrated in PBS buffer. Next, CTDnps complexes were further concentrated with 100 kDa MWCO ultra-centrifugal filters (Millipore) and collected in PBS buffer for long-term storage at 4°C.

### DLS analysis

DLS was performed to measure the hydrodynamic diameter of ST-AP205 recombinant proteins and CTDnps complexes, using a Zeta PALS (Malvern Instruments) at 25°C. All samples were centrifuged (15,000 × *g* for 20 min at 4°C) to remove aggregates, and diluted to 0.2 mg/mL in PBS. Each protein sample was loaded into a reusable cuvette, size distributions were recorded at 25°C with three scans of 10 s each, and the data were analyzed using software provided by Malvern Instruments.

### Transmission electron microscopic (TEM) analysis

The morphology of ST-AP205 recombinant proteins and CTDnps complexes was evaluated using TEM. For this, 10 µL of each sample was added to glow-discharged 300-mesh copper grids. After incubation for 30 s, excess sample was absorbed onto filter paper. Negative staining was immediately performed by adding 10 µL of uranyl acetate to the copper grids and incubating for 30 s. Liquid was removed using filter paper and grids were air-dried and imaged using a FEI Tecnai G2 transmission electron microscope (Thermo Fisher Scientific).

### Immunogenicity of CTDnps in mice and pigs

Fifteen female BALB/c mice, 6 weeks old, were randomly and equally allocated into three groups: CTD monomer group, CTDnps group, and PBS group. The immunization dose was 10 µg of protein monomer or the corresponding antigen-AP205 coupling to produce an equimolar dose of antigenic protein. Antigens were diluted in PBS and gently mixed 1:1 vol/vol with AddaVax adjuvant (Invivogen, Carlsbad, CA, USA) to reach a final volume of 200 µL. Each mouse received an intraperitoneal injection, two times, 4 weeks apart. Blood samples were collected 0, 2, 4, 6, and 8 weeks after primary immunization to detect PDCoV-specific IgG and NAb titers.

Six pregnant sows (Landrace) were purchased from a local farm and randomly and equally allocated into two groups: CTDnps group and PBS group. All sows were negative for PEDV, TGEV, and PDCoV antigens and antibodies before vaccination. At −42 and −14 days antepartum, sows were immunized intramuscularly with either 50 µg of CTD coupled to AP205 or given PBS. Blood samples were collected at −42, −14, 0, 30, and 60 days to detect titers of PDCoV-specific IgG and NAbs. In addition, milk samples were collected at 1, 3, and 5 days after farrowing to detect titers of PDCoV-specific sIgA and NAbs. Blood samples were collected from piglets at 1, 3, and 5 days of age to determine titers of PDCoV-specific IgG, sIgA, and NAbs.

Five 5-day-old piglets were randomly selected from each sow, fed pigger cream (Liprovit, Kampen, the Netherlands) four times a day, and challenged orally with the PDCoV HuN/2023 strain (4 mL × 10^5.0^ TCID_50_/mL per piglet). All piglets were negative for PEDV, TGEV, and PDCoV antigens before challenge. Challenged and unchallenged piglets were kept in separate rooms. All piglets were monitored for 7 days post-challenge, with daily collection of fecal swabs and assessment of clinical signs (e.g., diarrhea and vomiting). Diarrhea was graded as: 0 = solid; 1 = pasty; 2 = semiliquid; and 3 = liquid (64). Body weight was measured on the day of challenge and daily thereafter until necropsy. All piglets were necropsied at 7 days post-infection (dpi), and the intestinal tract was excised and photographed.

### iELISA

For analysis of PDCoV-specific IgG antibody titers in serum, ELISA plates were treated overnight at 4°C with 0.4 µg/well recombinant spike protein of PDCoV in PBS and washed five times with PBST. Plates were blocked with 100 µL of PBS containing 5% (wt/vol) skimmed milk at 37°C for 2 h. After washing plates three times with PBST, diluted serum (1:100, 100 µL/well) was added to each well in triplicate and incubated at 37°C for 30 min. Corresponding HRP-conjugated goat anti-mouse or pig IgG antibody (1:10,000 dilution in PBST) (KPL, MD, USA), HRP-conjugated goat anti-mouse IgG1 (Abcam, Cambridge, UK) or HRP-conjugated goat anti-mouse IgG2a (Abcam) was added and incubated at 37°C for 30 min. Reactions were performed at room temperature using TMB for 15 min and terminated with 2 M H_2_SO_4_. Absorbance was measured at 450 nm with a microplate spectrophotometer.

For analysis of PDCoV-specific sIgA antibody titers in milk, ELISA plates were treated overnight at 4°C with 0.1 µg/well recombinant spike protein of PDCoV in PBS and washed five times with PBST. Plates were blocked with 100 µL of PBS containing 5% (wt/vol) skimmed milk at 37°C for 2 h. After washing plates three times with PBST, diluted milk (1:40, 100 µL/well) was added to each well in triplicate and plates were incubated at 37°C for 1 h. HRP-conjugated goat anti-pig IgA antibody (1:30,000 dilution in PBST; Abcam) was added, followed by incubation at 37°C for 1 h. Reactions with TMB were performed at room temperature for 15 min and terminated with 2 M H_2_SO_4_. Absorbance was measured at 450 nm with a microplate spectrophotometer.

### VNT

Serum and milk samples were inactivated at 56°C for 30 min and diluted twofold, starting at 1:8. The ST cells were grown in 48-well culture plates to reach 90% confluency. Diluted serum was mixed with an equal volume of viral stock containing 500 TCID_50_ PDCoV in a 1.5 mL tube and incubated for 1 h at 37°C. The mixture was inoculated onto the pre-prepared ST cells monolayers in 48-well culture plates for 48 h at 37°C. The VNT titers were calculated according to the cytopathic effect and immunofluorescence assay. All diluted samples were tested in triplicate. For VNT_50_, titers were expressed as the highest serum or milk dilution resulting in 50% inhibition of PDCoV infection.

### Flow cytometry

At week 8, spleen lymphocytes of mice were isolated, cell concentration was adjusted to 1 × 10^7^ cells/mL, and 10 µL of FITC anti-mouse CD3 antibody, 10 µL of Violet 450 anti-mouse CD4 antibody, and 10 µL of PerCP/Cyanine5.5 anti-mouse CD8a antibody (Elabscience, Wuhan, China) were added to 200 µL of splenocytes for analysis of CD4^+^ and CD8^+^ T cells. Subsequently, stained cells were divided into two tubes (100 µL/tube). Then, 5 µL of APC anti-mouse LPAM-1 antibody (Elabscience) was added to one tube for examination of the CD4^+^α4β7^+^ T cells, whereas 5 µL of PE anti-human/mouse CD44 antibody and 5 µL of APC anti-mouse CD62L antibody (Elabscience) were added to the other tube for examination of Tcm cells. Stained cells were analyzed using a BD Accuri C6 Plus flow cytometer, with data analyzed using FlowJo software.

### Histopathology and immunofluorescence

Ileums collected from all piglets necropsied at 7 dpi were fixed in 10% neutral formalin, dehydrated, embedded, sectioned, and stained with H&E. To evaluate the distribution of PDCoV antigen in the small intestine, immunofluorescence was performed using PDCoV-N-specific monoclonal antibodies with FITC-conjugated goat anti-mouse IgG secondary antibodies (Abcam).

### Detection of dendritic cellular uptake and activation

DC2.4 or BMDCs were inoculated in 24-well plates (1 × 10^6^ cells per well) and cultured overnight at 37°C. Then CTD or CTDnps were added to pre-prepared wells, with a consistent molar dose of CTD antigen. PBS-added cells were used as a negative control. After 6 h of incubation, cells were harvested, washed three times with PBS, and immobilized for 20 min in 4% paraformaldehyde (Solarbio, Beijing, China) and then adhered onto poly-L-lysine-coated glass coverslips and permeabilized for 10 min with 0.1% Triton X-100 (Solarbio) at room temperature. After three washes with PBS, cells were exposed to the PDCoV CTD-specific monoclonal antibody, then incubated with a secondary antibody labeled with fluorescent dye, and mounted with ProLong Gold antifade reagent with 4′,6-diamidino-2-phenylindole (DAPI, Invitrogen) and examined with a confocal immunofluorescent microscope (Carl Zeiss, Germany).

For detection of activation, BMDCs were inoculated in 24-well plates (1 × 10^6^ cells per well) and cultured overnight at 37°C. Then CTD or CTDnps were added to pre-prepared wells, with a consistent molar dose of CTD antigen. PBS-added cells were used as a negative control. After 12 h of incubation, cells were harvested and washed three times with PBS. The cells were lysed in TransZol Up (Transgen, Beijing, China) according to the manufacturer’s instructions. Approximately 1 µg of RNA was reverse transcribed to cDNA synthesis using the EasyScript One-Step gDNA Removal and cDNA Synthesis SuperMix (Transgen) according to the manufacturer’s instructions. Expression of target genes was normalized to GAPDH expression. The delta-delta cycle threshold (CT) method was used to calculate relative abundance of *MHC II*, *CD80*, and *CD86* cDNA from CTD or CTDnps-added BMDCs relative to the PBS-added cell control. Primers are described in Table 1.

**TABLE 1:** Primers used for RT-qPCR

Gene	Sequence (5′−3′)
*PDCoV N*	F: CGCTTAACTCCGCCATCAA
	R: TCTGGTGTAACGCAGCCAGTA
*MHC II*	F: CCGAACACCAATGTACCTCCA
	R: GCCAGGTGACATTGACCACT
*CD80*	F: CAGCCTCAGCATGTGGGATT
	R: GTGTCCATCGATGCTCTAGGT
*CD86*	F: GTCGTTGTGTGTGGGATGGT
	R: TTCATGGACTTCTGCTCTGTTCT
*GAPDH*	F: AGGGCATCCTGGGCTACACT
	R: TCCACCACCCTGTTGCTGTA

### RT-qPCR

Fecal swabs and portions of small intestines were used for RNA extraction using a UE Body Fluid Viral DNA/RNA Miniprep Kit (Axygen, MA, USA) according to the manufacturer’s instructions. Then, the extracted RNA was tested for viral load of PDCoV using RT-qPCR. Specific primers targeting the PDCoV *N* gene are listed in Table 1. Three biological replicates were performed, each in technical triplicate. Each experiment included controls lacking template and lacking reverse transcriptase.

### Statistical analysis

Statistical significance was determined by two-tailed Student’s *t*-test or two-way ANOVA (GraphPad, Version 8.4.3); resulting “*P* values” were indicated as follows: ns, no significance; *, *P* < 0.05; **, *P* < 0.01; and ***, *P* < 0.001. Data were reported as mean ± SD of at least three independent experiments.

## Data Availability

The data that support the findings of this study are available from the corresponding author upon reasonable request.
